# Supplementary material for: Cellular state landscape and herpes simplex virus type 1 infection progression are connected
Source: Nat Commun. 2023 Jul 27;14:4515. doi: 10.1038/s41467-023-40148-6 (PMC10374626; doi:10.1038/s41467-023-40148-6)
Supplement: Supplementary file 1 — Supplementary Information [file 41467_2023_40148_MOESM1_ESM.pdf]

SUPPLEMENTARY INFORMATION

Cellular state landscape and herpes simplex virus type 1 infection progression are connected

Maija K. Pietilä[1,*], Jana J. Bachmann[1], Janne Ravantti[2], Lucas Pelkmans[3], and Cornel Fraefel[1,*]

[1] Institute of Virology, University of Zurich, Zurich, Switzerland
[2] Molecular and Integrative Biosciences Research Programme, University of Helsinki, Helsinki, Finland
[3] Department of Molecular Life Sciences, University of Zurich, Zurich, Switzerland
[*] Correspondence: maija.pietilae@uzh.ch (M.K.P.), cornel.fraefel@uzh.ch (C.F.)

SUPPLEMENTARY FIGURES

Supplementary Figure 1

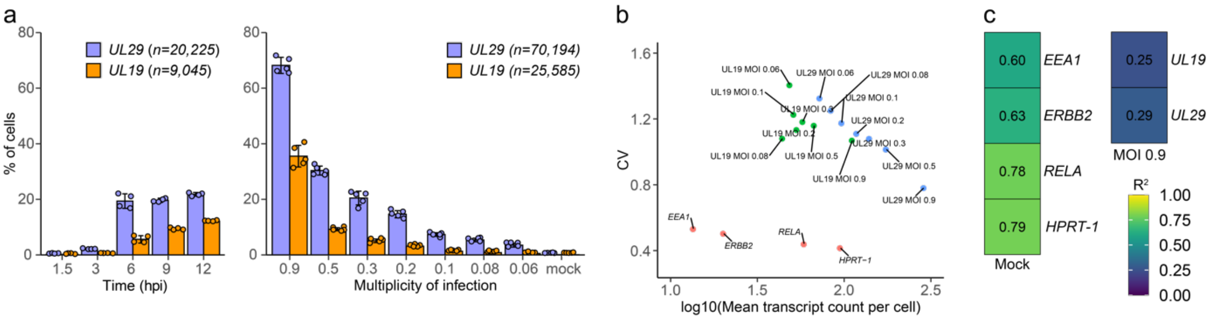

Supplementary Fig. 1. Related to Fig. 1: Heterogeneity in HSV-1 *UL29* and *UL19* transcript counts.

a Percentage of cells expressing *UL29* or *UL19* at MOI 0.3 at 1.5-12 hpi (left) or at multiplicities of infection (MOIs) 0.9-0.06 or in mock cells at 6 hpi (right). HeLa cells were infected with HSV-1 and stained for cytoplasmic *UL29* and *UL19* transcripts using smFISH. Data were acquired from two independent experiments and represent mean ± standard deviation among four (left) or five (right) individual replicate wells.

b Coefficient of variation (CV) of *UL29* and *UL19* at MOIs 0.9-0.06 at 6 hpi and of four cellular transcripts (*HPRT-1*, *ERBB2*, *EEA1*, and *RELA*) versus their mean transcript count (n=5 wells).

c Multiple linear regression (MLR) and explained variance (R^2). To predict cytoplasmic transcript abundance of *UL29* and *UL19* in HSV-1-infected HeLa cells (MOI 0.9) at 6 hpi as well as of four cellular transcripts in mock-infected HeLa cells, MLR was applied in a principal component (PC)-reduced space of the cell phenotypic state (331 single-cell features) from 12,500 cells per gene. The cell state was determined by quantifying DNA and protein intensity and texture, morphology features, number of neighbors, location in the population, local cell density, and cell-cycle phase of single cells. R^2 shown represents mean of five predictions.

Supplementary Figure 2

a

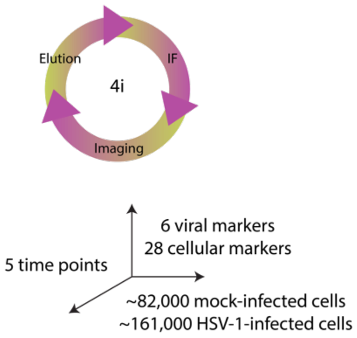

b

Viral mRNA

Early:
- *UL29*

Late:
- *UL19*

Viral proteins

Immeadiate early:
- ICP0, ICP4, ICP27

Early:
- ICP8

Late:
- ICP5, VP16

Transcription

RNAP II
RNAP II S5P
RNAP II S2P
p-CDK9
HCFC1
H3
H3K4me3

Signaling

p-ERK
p-Akt
p-EGFR
EGFR
CTNNB1

Subcellular compartments and cytoskeleton

C23
SRSF2
NUPS
DDX6
CRT
Hsp60
GM130
TGN46
EEA1
Beta-actin
Alpha-tubulin

Cell cycle

PCNA

Antiviral response

SP100
IRF3
NRF2
Cleaved caspase 3

DNA and protein

DAPI
Succinimidyl-ester

c

Neighborhood

9 features
- Number of neighbors
- Fraction touching neighbors
- Distance to cell islet edge
- At cell islet edge
- Local cell density with radii of 100, 200, 300, 400, and 500 pixels

Morphology

184 features

For cell, nucleus, and cytoplasm:
- Area
- Perimeter
- Eccentricity
- Extent
- Convexity
- Circularity
- Roundness
- Elongation
- Equivalent diameter
- Major axis length
- Minor axis length
- Maximum radius
- Mean radius
- Zernike moments

Staining

180 intensity features

For cell, nucleus, and cytoplasm:
- Mean and sum intensity of 28 cellular markers, DAPI, and succinimidyl-ester

2,758 texture features

For 28 cellular markers, DAPI, and succinimidyl-ester in cell, nucleus, and cytoplasm:
- Gabor frequency (Kernel size: 1, 5, 10)
- Hu moments (0, 1)
- Haralick textures (Kernel size: 2, 10)
  Angular second moment, contrast, correlation, sum of squares, inverse difference moment, sum average, sum variance, sum entropy, entropy, difference variance, difference entropy, information measures of correlation 1 and 2

4 count features
- Splicing speckles, nucleoli, PML bodies, and P bodies

1 cell-cycle feature
- G1, S, or G2 (mitotic cells were excluded)

Multicellular — **Cellular** — **Subcellular**

d

Neighborhood

1 feature
- Neighbor infection score

Staining

48 intensity features

For cell, nucleus, and cytoplasm:
- Mean and sum intensity of 8 viral markers

694 texture features

For 8 viral markers in cell, nucleus, and cytoplasm:
- Gabor frequency (Kernel size: 1, 5, 10)
- Hu moments (0, 1)
- Haralick textures (Kernel size: 2, 10)
  Angular second moment, contrast, correlation, sum of squares, inverse difference moment, sum average, sum variance, sum entropy, entropy, difference variance, difference entropy, information measures of correlation 1 and 2

1 count feature
- Viral replication compartments

6 spot count features

For cell, nucleus, and cytoplasm
- UL29 and UL19

Supplementary Fig. 2. Related to Fig. 1: Single-cell features extracted from the smFISH + 4i experiment.
a Schematic of iterative indirect immunofluorescence imaging (4i) workflow applied to mock- and HSV-1-infected HeLa cells after smFISH.
b Markers used in smFISH and 4i.
c 3,136 multicellular, cellular, and subcellular single-cell features extracted from the smFISH + 4i experiment.
d 750 viral single-cell features extracted from two viral mRNAs and 6 viral proteins used in the smFISH + 4i experiment.

Supplementary Figure 3

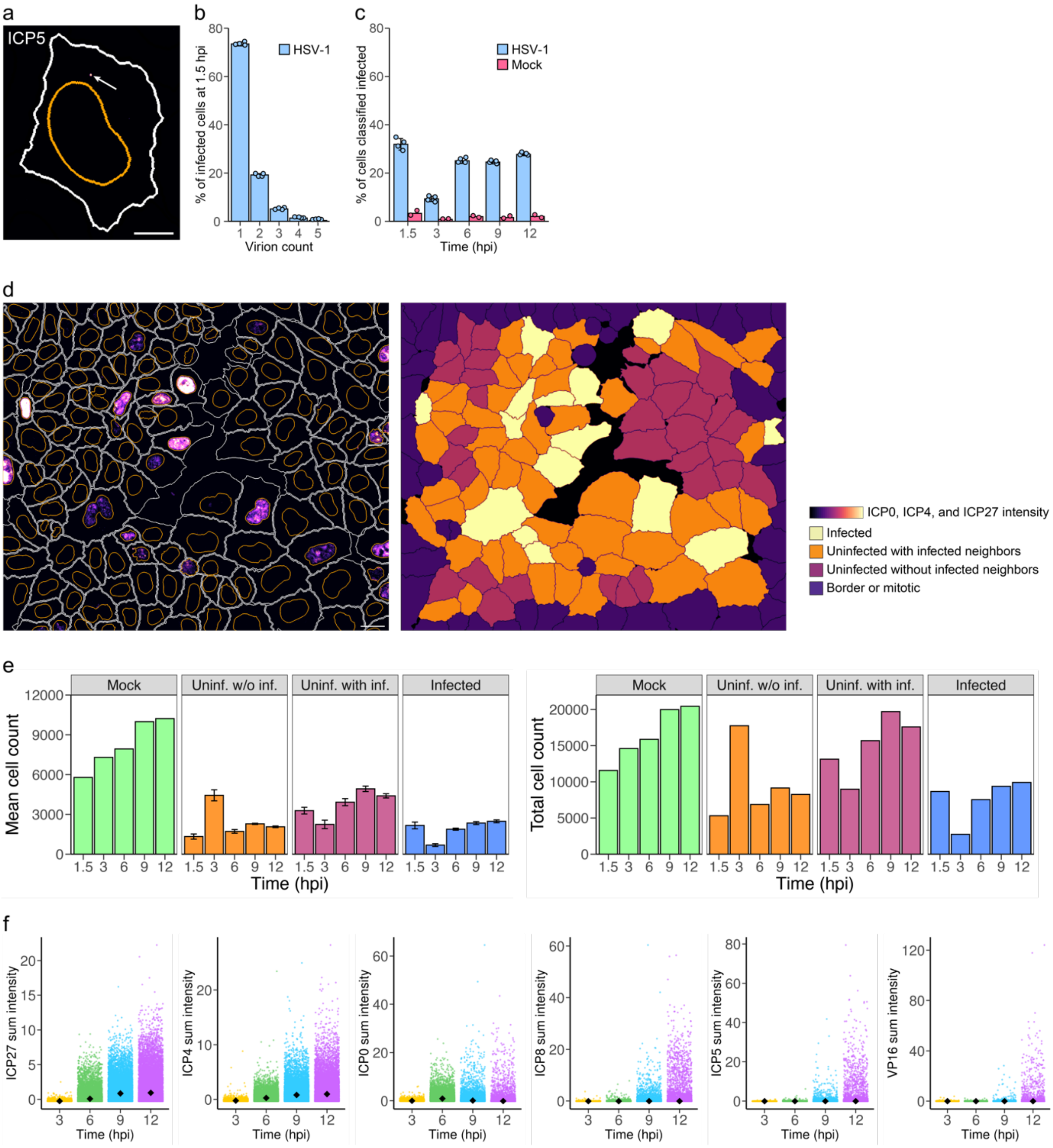

Supplementary Fig. 3. Related to Fig. 1: Quantification of infection and heterogeneity in the smFISH + 4i experiment.

a HSV-1-infected HeLa cell stained for the major capsid protein ICP5 at 1.5 hpi. White arrow points to an ICP5-positive spot, and orange and white lines indicate nucleus and cell outlines, respectively. Scale bar, 10 μm.

b Distribution of single-cell ICP5-virion counts at 1.5 hpi. Only cells classified as infected are considered. Data represent mean ± standard deviation among four individual replicate wells. Cells with more than 5 virions are not shown.

c Percentage of infected cells at MOI 0.3 at 1.5-12 hpi. Mock or HSV-1-infected HeLa cells were stained for viral immediate early proteins ICP0, ICP4, and ICP27, and for late protein

ICP5. At 1.5 hpi, cells were classified as infected if they contained at least one ICP5-positive spot representing incoming virions. At 3-12 hpi, cells were classified as infected if they expressed one or more of ICP0, ICP4, and ICP27. Data represent mean ± standard deviation among four (HSV-1) or mean among two (mock) replicate wells.
d Cell subpopulations after infection. Left: ICP0, ICP4, and ICP27 staining of HSV-1-infected HeLa cells at 12 hpi. Nucleus and cell outlines as in **a**. Scale bar, 25 μm. Right: cell classification using computer vision.
e Cell counts. Data represent mean ± standard deviation (left) or sum counts (right) among four (HSV-1) or mean (left) or sum counts (right) among two (mock) replicate wells. Labels: Uninf. w/o inf. = Uninfected cells without infected neighbors, Uninf. with inf. = Uninfected cells with infected neighbors.
f Distribution of normalised single-cell intensities of viral 4i markers in infected HeLa cells at 3-12 hpi. Black diamond indicates population median at each time point.

Supplementary Figure 4

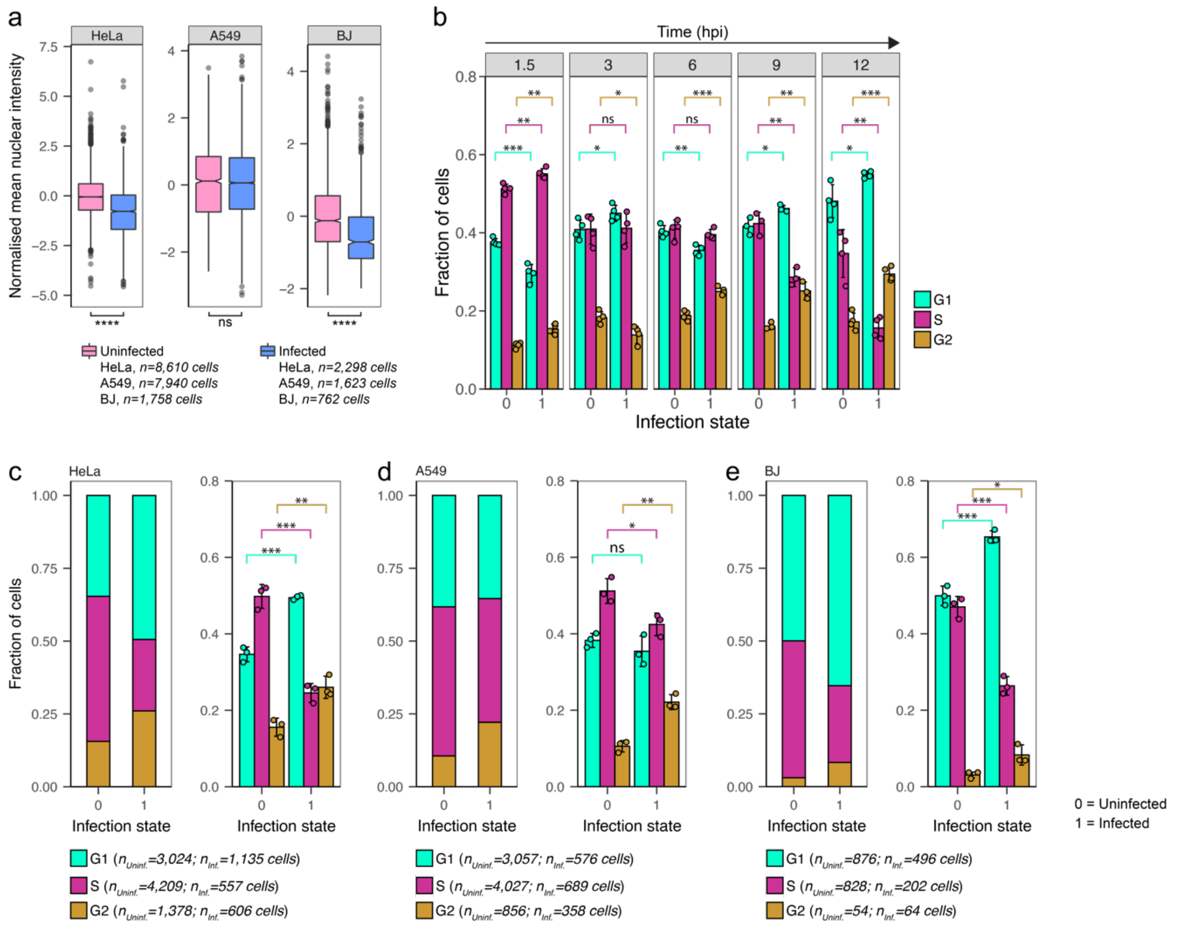

Supplementary Fig. 4. Related to Fig. 2: Cell cycle and cell size during HSV-1 infection.
a Validation of mean nuclear intensity of PCNA in uninfected and infected HeLa, A549 and BJ cells. Cells were infected with HSV-1 (MOI 0.3) and stained for ICP27 and PCNA at 12 hpi. Data are from one experiment. Cell counts are indicated in plots (n=3 wells). Cells were classified into uninfected and infected based on ICP27 expression. PCNA distributions were compared using pairwise two-sided Kolmogorov-Smirnov (KS) test. Boxplots are as follows: median count of cell population (central mark), 25th percentile (Q1; lower hinge), 75th percentile (Q3; upper hinge), smallest observation greater than or equal to Q1-1.5*interquartile range (lower whisker), largest observation less than or equal to Q3+1.5*interquartile range (upper whisker), and outliers (points).
b Cell-cycle phase of uninfected (0) or infected (1) HeLa cells from the smFISH + 4i experiment. Data represent mean ± standard deviation among three or four replicate wells. The same data are also presented in Fig. 2c. Number of cells in different cell-cycle phases was compared between uninfected and infected cells within a time point using two-sided unpaired two-sample t-test.
c-**e** Validation of cell-cycle classification in HeLa (**c**), A549 (**d**), and BJ (**e**) cells. Data represent mean ± standard deviation among three replicate wells. Data are from the same experiment as in **a**, and cell-cycle phases of uninfected (0) and infected (1) cells are presented as a stacked bar plot (left) and grouped bar plot (right). Cell counts are indicated in plots (n=3 wells). Number of cells in different cell-cycle phases was compared as in **b**.

In **a**-**e**: * $p < 0.05$, ** $p < 0.01$, *** $p < 0.001$, **** $p < 0.0001$, ns = not significant.

Supplementary Figure 5

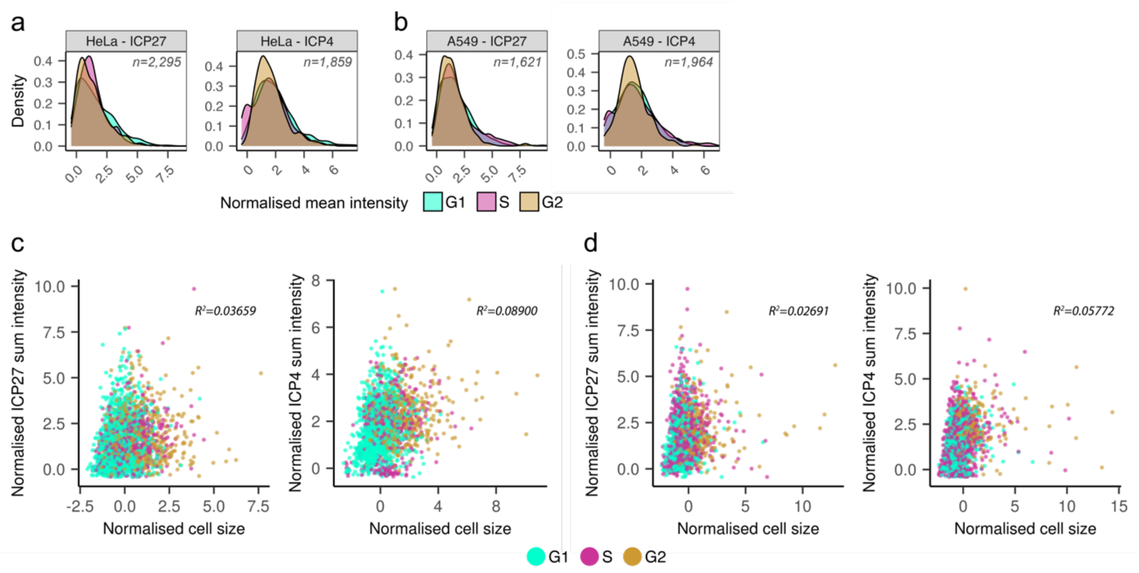

Supplementary Fig. 5. Related to Fig. 2: Cell cycle and cell size and HSV-1 gene expression.
a, **b** Single-cell ICP27 and ICP4 mean intensities in G1, S, and G2-phase HeLa (**a**) and A549 (**b**) cells expressing either ICP27 or ICP4. Cells were infected with HSV-1 (MOI 0.3) and stained for ICP27 and PCNA or for ICP4 and PCNA at 12 hpi. Data are from one experiment, and data for ICP27 is from the same experiment as data in Supplementary Fig. 4a. Cell counts are indicated in plots (n=3 wells). 0.1-99.9th percentiles of marker intensities are shown in the density plots.
c, **d** Single-cell ICP27 and ICP4 sum intensities versus cell size in ICP27 or ICP4-expressing HeLa (**c**) and A549 (**d**) cells. Data are from the same experiments as shown in **a** and **b**. Inset: R^2 of robust linear regression models fit to the single-cell data.

Supplementary Figure 6

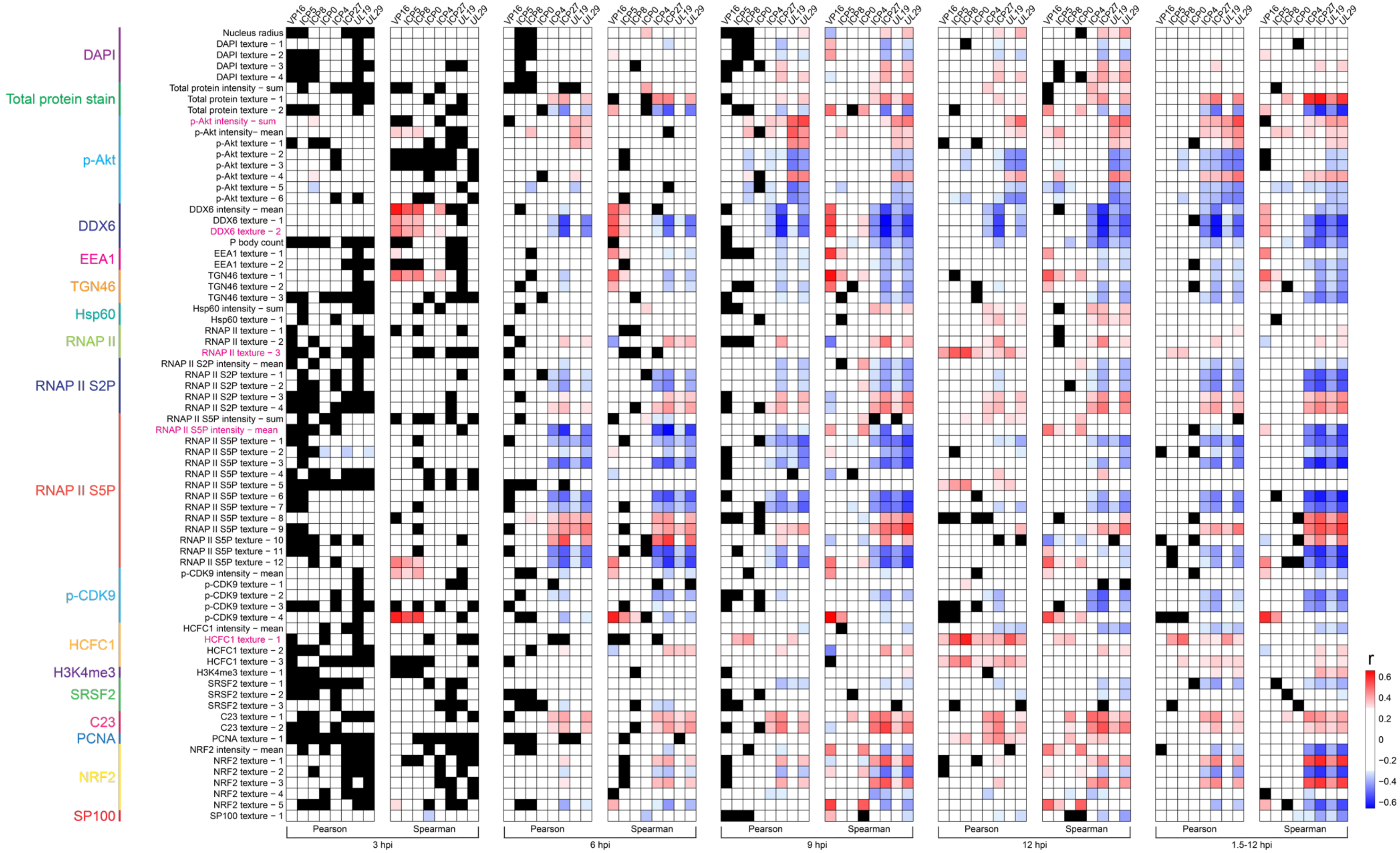

1

Supplementary Fig. 6. Related to Fig. 3: Correlation of cellular features with HSV-1 gene expression.

Pairwise correlation of viral and cellular features in infected HeLa cells from the smFISH + 4i experiment. Pearson correlation coefficient (r) was calculated, and only those cellular features are shown that gave Pearson r >= 0.3 or <= -0.3 with one or several viral features, and only correlations with r >= 0.3 or <= -0.3 are colored red or blue, respectively. Black indicates correlations with p value >= 0.05. Spearman's rank (r) was calculated for the same correlation pairs. Cellular features used as predictors in Supplementary Fig. 7b are highlighted in magenta. Full feature names are listed in Supplementary Data 3. For ICP27, ICP4, ICP0, VP16, ICP8, and ICP5, normalised sum intensities were used. For *UL29* and *UL19*, spot counts were used.

See also Supplementary Fig. 7 and Supplementary Data 3.

Supplementary Figure 7

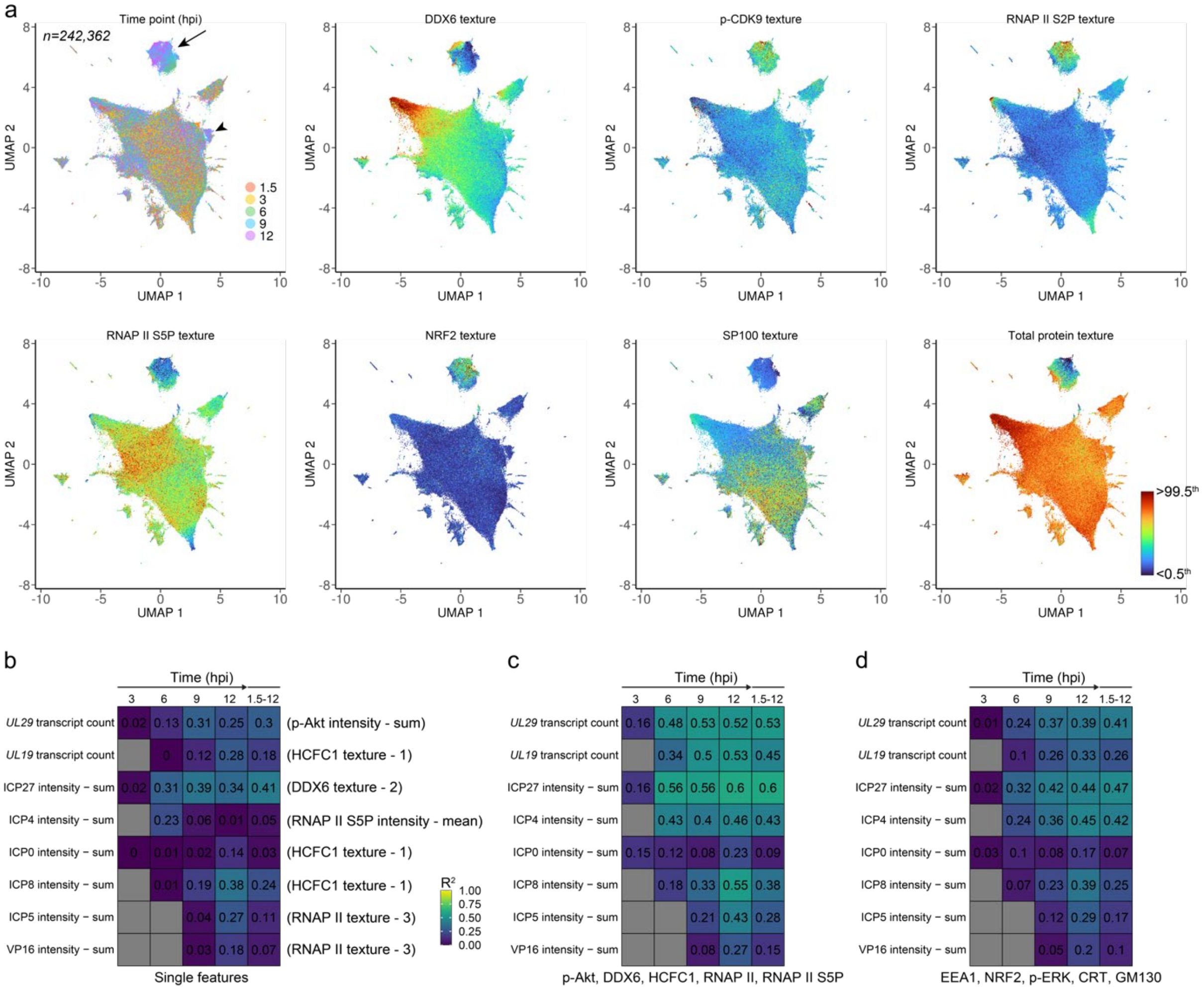

Supplementary Fig. 7. Related to Fig. 3: Cellular features and HSV-1 infection heterogeneity.

a UMAP of mock- and HSV-1-infected HeLa cells (as in Fig. 3a) colored by time point (hpi) or by the following Haralick texture features: DDX6, difference entropy (Kernel size: 2) in cell; p-CDK9, entropy (Kernel size: 10) in nucleus; RNAP II S2P, information measures of correlation (Kernel size: 2) in nucleus; RNAP II S5P, sum entropy (Kernel size: 2) in nucleus; NRF2, angular-second moment (Kernel size: 2) in nucleus; SP100, sum variance (Kernel size: 2) in nucleus; total protein staining, correlation (Kernel size: 10) in nucleus. Scale bar: lower limit is <0.5th and upper limit is >99.5th percentile of the values. Arrow, a cluster formed by infected cells at 6-12 hpi. Arrowhead, a cluster formed by uninfected cells with infected neighbors at 9 and 12 hpi. Data are from two (mock) or four (HSV-1 infection) individual replicate wells per time point. Cell count is indicated in the first UMAP.

b Linear regression and explained variance (R^2). For each viral marker, a top Pearson correlator across all time points was used as a single predictor in linear regression to model viral gene expression in infected HeLa cells from the smFISH + 4i experiment. Predictors are indicated in parentheses (see also Supplementary Data 3). R^2 shown represents mean of four predictions. Grey, less than 250 cells expressing the corresponding viral marker.

c Multilinear regression and explained variance (R^2). A PC-reduced space of 495 single-cell features from p-Akt, DDX6, HCFC1, RNAP II, and RNAP II S5P stainings was used in MLR to

predict viral gene expression in infected HeLa cells. R^2 shown represents mean of four predictions. Grey, less than 250 cells expressing the corresponding viral marker.
d MLR and explained variance (R^2). As in **c** but using single-cell features from EEA1, NRF2, p-ERK, CRT, and GM130 stainings.

Supplementary Figure 8

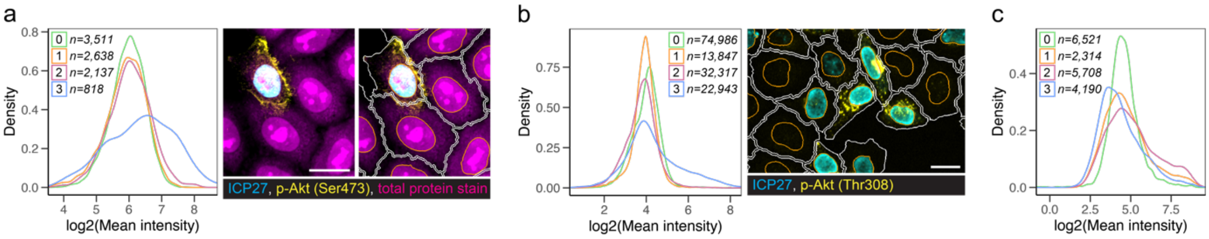

Supplementary Fig. 8. Related to Fig. 5: Activation of Akt and ERK in HeLa cells.
a Validation of Akt (Ser473) activation in HeLa cells. Cells were mock infected or infected with HSV-1 (MOI 0.3) and stained for ICP27 and p-Akt (Ser473) at 12 hpi. Left: distribution of log_2-transformed single-cell mean intensity of p-Akt (Ser473) in four cell subpopulations: mock cells (0), uninfected cells without infected neighbors (1), uninfected cells with infected neighbors (2), and infected cells (3). Data are from one experiment. Cell counts are indicated in a plot (n_{mock}=1 well and n_{HSV-1}=2 wells). Right: ICP27, p-Akt (Ser473) and total protein staining of HSV-1-infected HeLa cells. Orange and white lines indicate nucleus and cell outlines, respectively. Scale bar, 25 μm. p-Akt (Ser473) increase in subpopulation 2 could be due to slight missegmentation of cell border as p-Akt (Ser473) signal was enriched to the cell border.
b p-Akt (Thr308) after infection. HeLa cells were mock infected or infected with HSV-1 (MOI 0.3) and stained for ICP27 and p-Akt (Thr308) at 12 hpi. Left panel: distribution of log_2-transformed single-cell mean intensity of p-Akt (Thr308) in four HeLa cell subpopulations (as in **a**). Data are from two independent experiments (n=3 wells per condition per experiment). Cell counts are indicated in a plot. Right panel: ICP27 and p-Akt (Thr308) staining of HSV-1-infected HeLa cells. Nucleus and cell outlines as in **a**. Scale bar, 25 μm.
c Validation of ERK activation in HeLa cells. Cells were mock infected or infected with HSV-1 (MOI 0.3) and stained for ICP27 and p-ERK at 12 hpi. Distribution of log_2-transformed single-cell mean intensity of p-ERK in four cell subpopulations as in **a**. Data are from one experiment. Cells counts are indicated in the plot (n_{mock}=1 well and n_{HSV-1}=2 wells).
0.1-99.9th percentiles of marker intensities are shown in the density plots.

Supplementary Figure 9

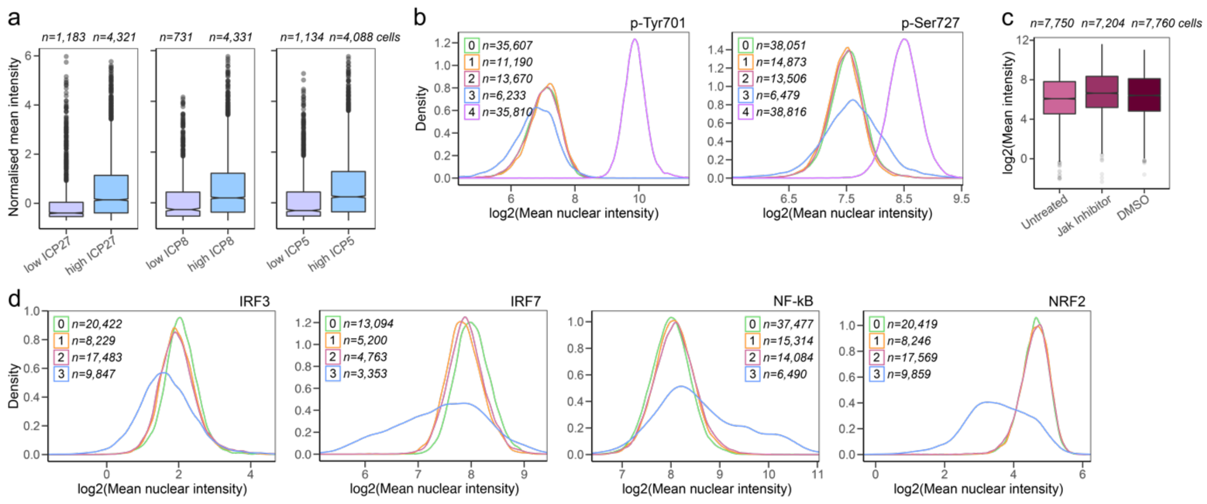

Supplementary Fig. 9. Related to Fig. 5: Phosphorylation of ERK in HeLa cells.

a Comparison of p-ERK mean intensity in uninfected HeLa cells next to infected cells at 12 hpi from the smFISH + 4i experiment (n = 4 wells). Uninfected cells with infected neighbors were divided into two groups: All infected neighbors have low levels of ICP27, ICP8, or ICP5 (indicated by “low”) or at least one infected neighbor has high levels of ICP27, ICP8, or ICP5 (indicated by “high”). Low-expressing cells: normalised mean intensity of ICP27, ICP8, or ICP5 < 20th percentile. High-expressing cells: normalised mean intensity of ICP27, ICP8, or ICP5 > 80th percentile. Cell counts are indicated above plots. Boxplots definitions as in Supplementary Fig. 4a.

b Density plots summarise log_2-transformed mean nuclear intensity of STAT1 p-Tyr701 and p-Ser727 in mock- and HSV-1-infected HeLa cell subpopulations at 12 hpi (MOI 0.3): mock cells (0), uninfected cells without infected neighbors (1), uninfected cells with infected neighbors (2), infected cells (3), and IFN-γ-treated mock cells (4). Data are from one experiment with two individual replicate wells per condition, and cells counts are indicated in plots.

c Boxplots summarise single-cell, log_2-transformed mean intensity of p-ERK in uninfected HeLa cells with infected neighbors (subpopulation 2 cells, cell counts are indicated above a plot): untreated or treated with 10 µM Jak inhibitor or 0.1% DMSO. Cells were pre-treated with the inhibitor or DMSO for 3 h before infection, and then the inhibitor or DMSO was added again at 1.5 hpi. Data are from one experiment with two individual replicate wells per condition. Boxplots definitions as in Supplementary Fig. 4a.

d Distribution of single-cell, log_2-transformed mean nuclear intensity of IRF3, IRF7, NF-κB, and NRF2 in mock HeLa cells and three HeLa cell subpopulations after HSV-1 infection (MOI 0.3) at 12 hpi. IRF7 and NF-κB data are from one experiment with 2 wells per condition. IRF3 and NRF2 data are from the smFISH + 4i experiment (two wells per mock and four wells per HSV-1 infection). Subpopulations as in **b**. Cells counts are indicated in plots.

$0.1\text{-}99.9^{th}$ percentiles of marker intensities are shown in the density plots, except for IRF3 in **d** that is shown in $0.2\text{-}99.8^{th}$ percentiles.

Supplementary Figure 10

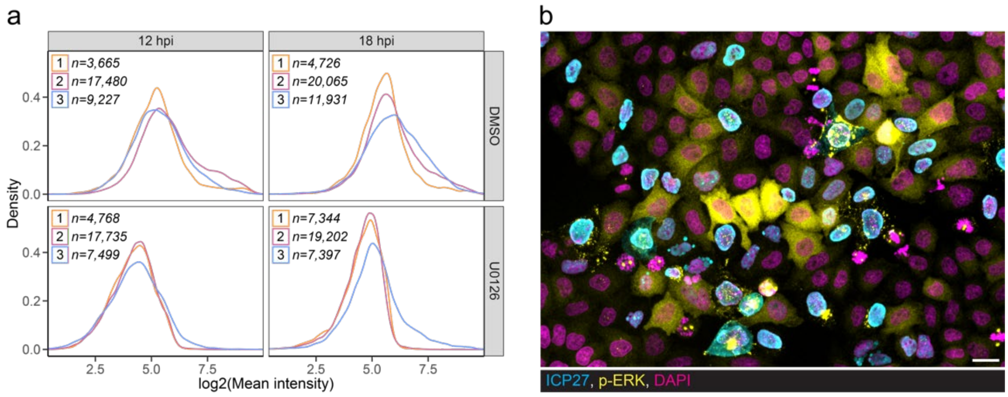

Supplementary Fig. 10. Related to Fig. 5: Inhibition of ERK phosphorylation in HeLa cells. **a** Density plots summarise log_2-transformed, single-cell mean intensity of p-ERK in HeLa cell subpopulations after HSV-1-infection (MOI 0.3) at 12 and 18 hpi (n=3 wells). Subpopulations: uninfected cells without infected neighbors (1), uninfected cells with infected neighbors (2), infected cells (3). Cells were treated with 50 μM U0126 or 0.1% DMSO at 1.5 hpi. Cell counts are indicated in plots. Quantification of the same cells is also shown in Fig. 5d. 0.1-99.9th percentiles of marker intensities are shown in the density plots. **b** ICP27, p-ERK, and DAPI staining of HSV-1-infected HeLa cells (MOI 0.3) at 18 hpi. Scale bar, 25 μm.

Supplementary Figure 11

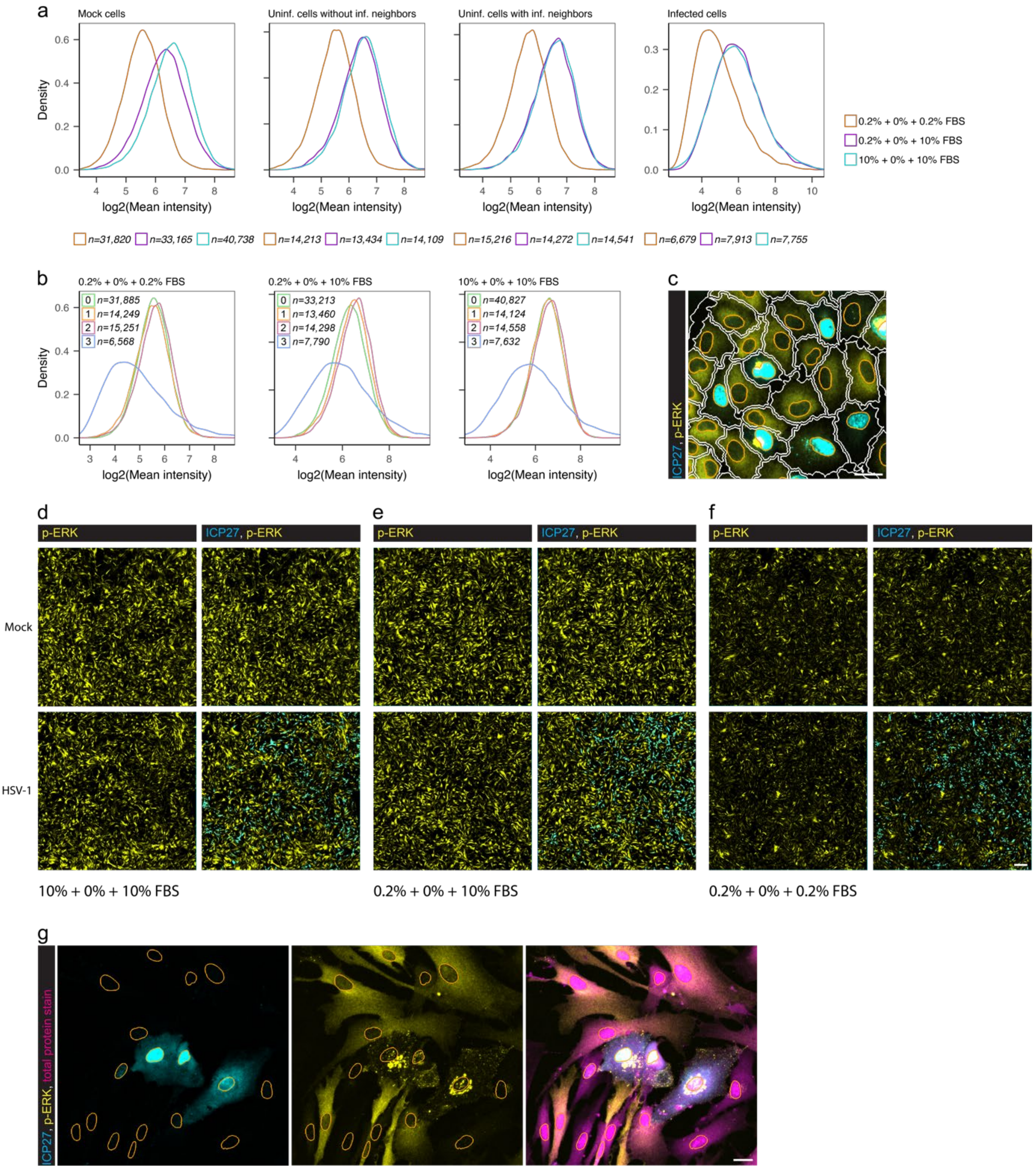

Supplementary Fig. 11. Related to Fig. 5: Phosphorylation of ERK in A549 and BJ cells.
a p-ERK levels in A549 cells and cell culture medium. Cells were mock infected or infected with HSV-1 (MOI 0.3) and stained for ICP27 and p-ERK at 12 hpi. Cells were maintained in the presence of 10% FBS, infected in the absence of serum, and then grown in the presence of 10% FBS (10% + 0% + 10% FBS), cells were serum starved for 12 h before infection by incubating them in 0.2% FBS, infected in the absence of serum, and then grown in the presence of 10% FBS (0.2% + 0% + 10% FBS), or cells were serum starved for 12 h before infection by incubating them in 0.2% FBS, infected in the absence of serum, and then grown in the presence of 0.2% FBS (0.2% + 0% + 0.2% FBS). Distribution of log_2-transformed single-

cell mean intensity of p-ERK is shown in three growth conditions for four cell subpopulations. Data are from one experiment with three individual replicate wells per condition. Cell counts are indicated in plots.
b HSV-1 infection and p-ERK levels in A549 cells. Data are from the same experiment as in **a**. Distribution of log_2-transformed single-cell mean intensity of p-ERK is shown in four cell subpopulations for three growth conditions. Cell counts are indicated in plots.
c p-ERK and ICP27 staining of HSV-1-infected A549 cells at 12 hpi. No serum starvation was performed. A representative image from the same experiment as in **a** is shown. Orange and white lines indicate nucleus and cell outlines, respectively. Scale bar, 25 μm.
d-**f** p-ERK levels in BJ cells and cell culture medium. Cells were mock infected or infected with HSV-1 (MOI 0.3) and stained for ICP27 and p-ERK at 12 hpi. Cells were maintained as described in **a**. Scale bar, 250 μm.
g p-ERK and ICP27 staining of HSV-1-infected BJ cells at 12 hpi. No serum starvation was performed. A representative image from the same experiment as in **d** is shown. Nucleus outlines are indicated in orange. Scale bar, 25 μm.

Supplementary Figure 12

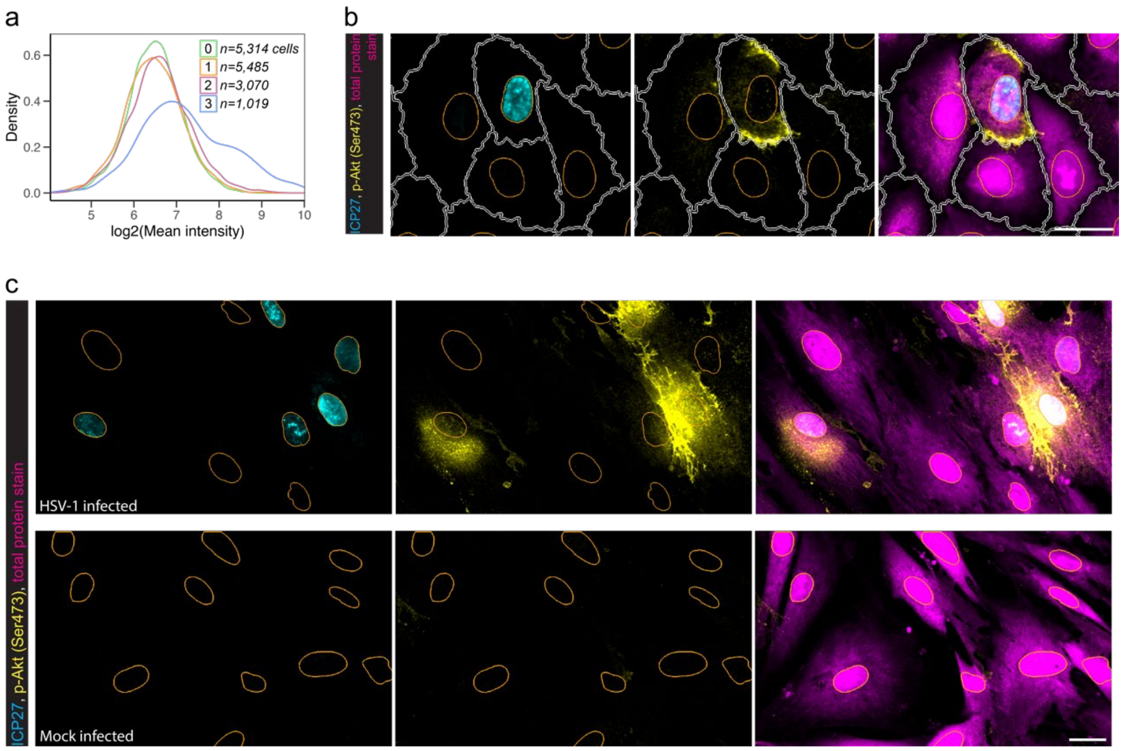

Supplementary Fig. 12. Related to Fig. 5: Phosphorylation of Akt in A549 and BJ cells.
a Validation of Akt (Ser473) activation in A549 cells. Cells were mock infected or infected with HSV-1 (MOI 0.3) and stained for ICP27 and p-Akt (Ser473) at 12 hpi. Density plot shows distribution of log_2-transformed single-cell mean intensity of p-Akt (Ser473) in four cell subpopulations: mock cells (0), uninfected cells without infected neighbors (1), uninfected cells with infected neighbors (2), and infected cells (3). Data are from one experiment. Cell counts are indicated in a plot (n_{mock}=1 well and $n_{HSV\text{-}1}$=2 wells). 0.1-99.9th percentiles of marker intensities are shown in the density plot.
b ICP27, p-Akt (Ser473) and total protein staining of HSV-1-infected A549 cells. Orange and white lines indicate nucleus and cell outlines, respectively. Scale bar, 25 μm.
c Validation of Akt (Ser473) activation in BJ cells. Cells were mock infected (lower row) or infected with HSV-1 (MOI 0.3; upper row) and stained for ICP27 and p-Akt (Ser473) at 12 hpi. Nucleus outlines are indicated in orange. Scale bar, 25 μm.

Supplementary Figure 13

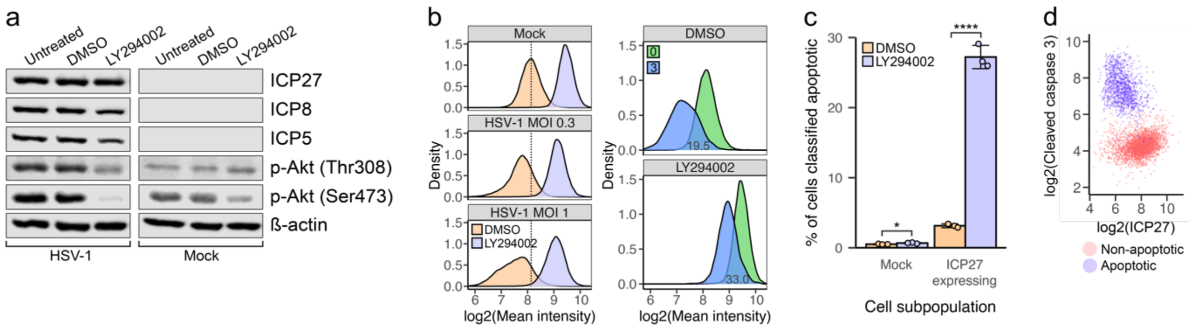

Supplementary Fig. 13. Related to Fig. 6: Inhibition of Akt phosphorylation.

a HeLa cells were mock infected or infected with HSV-1 (MOI 1) and treated with 0.1% DMSO or 50 µM LY294002 at 1.5 hpi. Cells were collected at 12 hpi and stained for ICP27, ICP8, ICP5, p-Akt (Thr308), p-Akt (Ser473), and β-actin using Western blotting. Representative blots are shown, and quantification from three biologically independent experiments is presented in Fig. 6b.

b Validation of 4E-BP staining in HeLa cells. Density plots summarise single-cell mean intensity of 4E-BP after mock or HSV-1 infection in HeLa cells at 12 hpi from three individual replicate wells (mock n_{DMSO}=28,122 and $n_{LY294002}$=20,876; HSV-1 MOI 0.3 n_{DMSO}=28,220 and $n_{LY294002}$=21,862; HSV-1 MOI 1 n_{DMSO}=23,878 and $n_{LY294002}$=20,632 cells). Cells were treated with DMSO or LY294002 at 1.5 hpi, and 4E-BP and ICP27 were detected by immunofluorescence imaging at 12 hpi. Right: mock cells (0) and ICP27-expressing cells from HSV-1 infection using MOI 0.3 (3) are compared. The overlapped estimated area of two distributions is indicated in the overlapping area as a mean percentage calculated from two biologically independent experiments (n=3 wells per experiment). Data from a biological replicate are shown in Fig. 6c.

c Validation of apoptosis in HeLa cells. Cells were treated with DMSO or LY294002 at 1.5 hpi, and cleaved caspase 3 and ICP27 were detected by immunofluorescence imaging at 12 hpi. Mock cells (n_{DMSO}=43,663 and $n_{LY294002}$=27,086 cells) and ICP27-expressing cells from HSV-1-infection using MOI 0.3 (n_{DMSO}=5,426 and $n_{LY294002}$=4,501 cells). Data are from one experiment and represent mean ± standard deviation among three replicate wells (Fig. 6d shows data from a biologically independent experiment). Percentage of apoptotic cells was compared between the treatments for each cell subpopulation by one-sided unpaired two-sample t-test. * $p < 0.05$, ** $p < 0.01$, *** $p < 0.001$, **** $p < 0.0001$.

d Mean intensity of cleaved caspase 3 as a function of ICP27 in the LY294002-treated, ICP27-expressing HeLa cells (MOI 0.3) at 12 hpi ($n_{non\text{-}apoptotic}$=3,272 and $n_{apoptotic}$=1,229 cells). Data are from the same experiment as in **c**.

0.1-99.9th percentiles of marker intensities are shown in the density plots.

Supplementary Figure 14

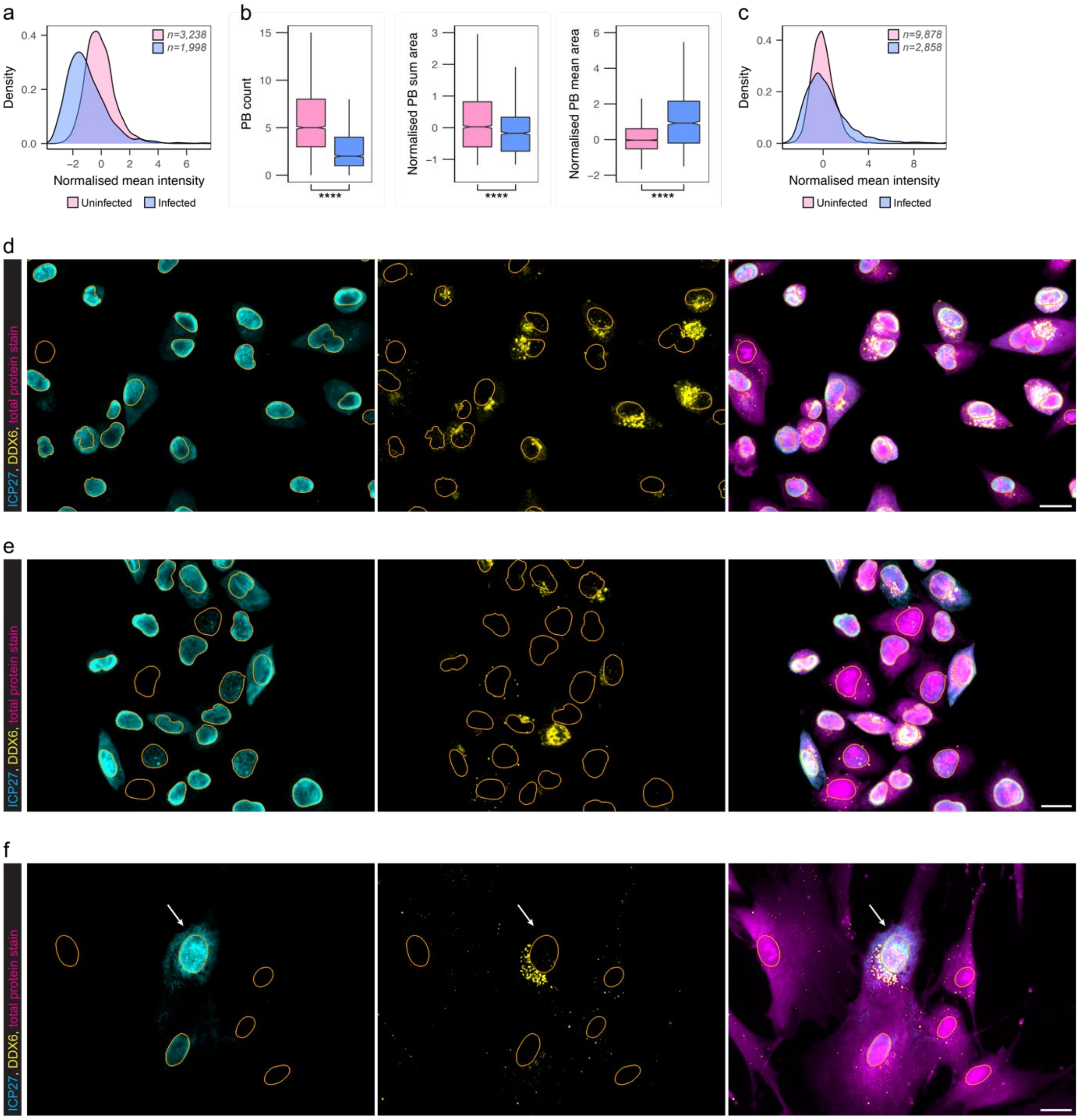

Supplementary Fig. 14. Related to Fig. 7: Loss of P bodies.

a Validation of DDX6 degradation in HeLa cells. Cells were infected with HSV-1 (MOI 0.3) and stained for DDX6 and ICP27 at 12 hpi. Density plot shows distributions of mean intensity of DDX6 in uninfected and HSV-1-infected cells. Data are from one experiment with three individual replicate wells. Inset: cell counts.

b Validation of PB transformation in HeLa cells. Boxplots summarise single-cell PB counts as well as PB sum and mean area in single uninfected and infected HeLa cells. Data are from the same experiment as in **a**. Distributions were compared using pairwise two-sided KS test: * $p < 0.05$, ** $p < 0.01$, *** $p < 0.001$, **** $p < 0.0001$. Boxplots definitions as in Supplementary Fig. 4a but outliers are omitted for clarity.

c Distributions of mean intensity of DDX6 in uninfected and HSV-1-infected (MOI 0.3) A549 cells from two independent experiments with 2 or 3 wells per experiment. Data from the same experiments are also shown in Fig. 7g. Inset: cell counts.
d DDX6 and ICP27 staining of HSV-1-infected (MOI 1) A549 cells at 12 hpi. A representative image from one experiment is shown (n=3 wells). Nucleus outlines are indicated in orange. Scale bar, 25 μm.
e DDX6 and ICP27 staining of HSV-1-infected (MOI 1) HeLa cells at 12 hpi. A representative image from one experiment is shown (n=3 wells). Nucleus outlines as in **d**. Scale bar, 25 μm.
f DDX6 and ICP27 staining of HSV-1-infected (MOI 0.3) BJ cells at 12 hpi. A representative image from two independent experiments is shown (n=1 or 3 wells per experiment). Arrow, ICP27-expressing cell with aggregated perinuclear DDX6 staining. Nucleus outlines as in **d**. Scale bar, 25 μm.
0.1-99.9th percentiles of marker intensities are shown in the density plots.

Supplementary Figure 15

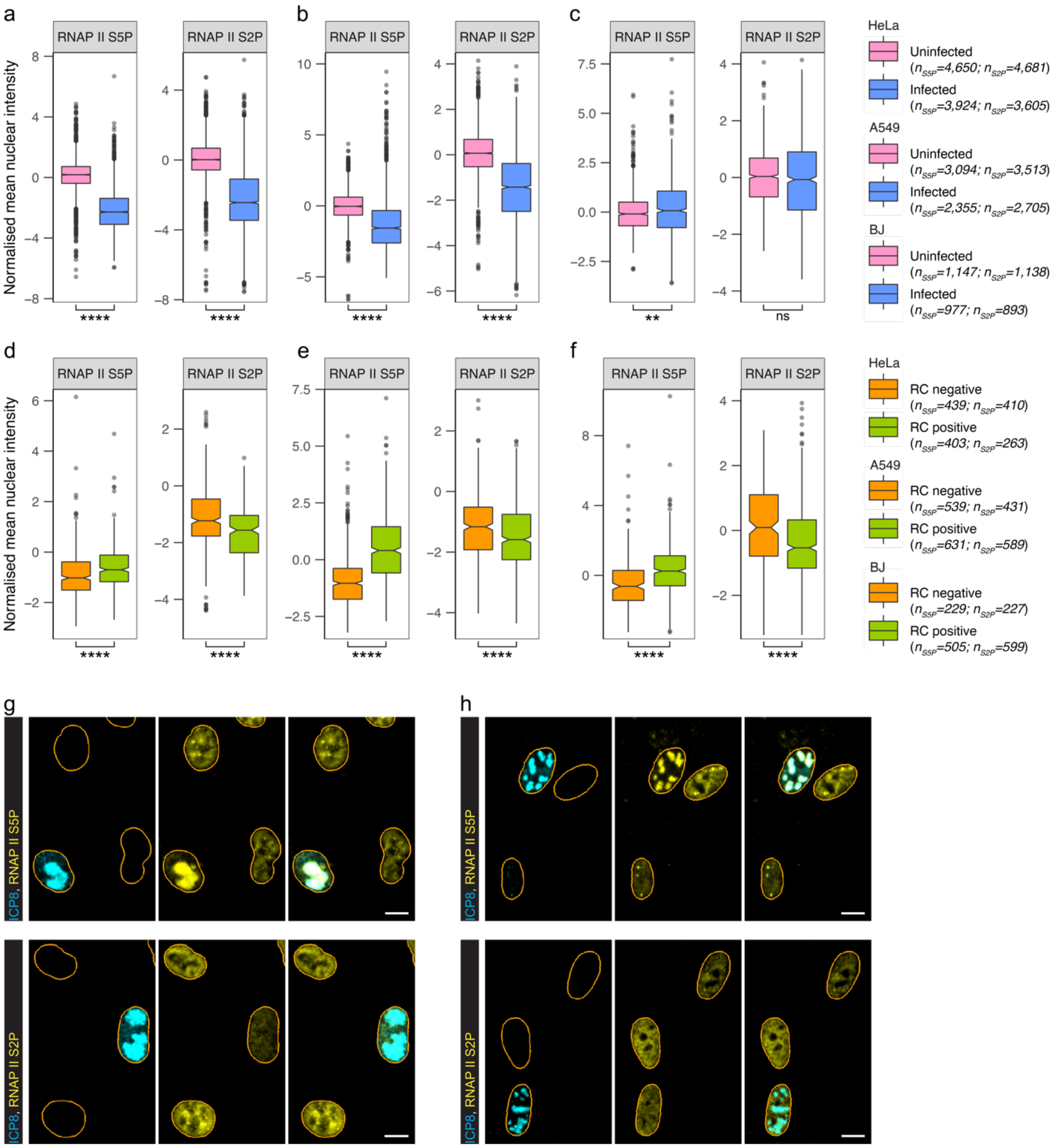

Supplementary Fig. 15. Related to Fig. 8: Phosphorylation changes of RNAP II during HSV-1 infection in HeLa, A549 and BJ cells.
a-**c** Validation of HSV-1-induced RNAP II phosphorylation changes in HeLa (**a**), A549 (**b**) and BJ (**c**) cells. Cells were infected with HSV-1 (MOI 0.3) and stained for ICP27 and RNAP II S5P or for ICP27 and RNAP II S2P at 12 hpi. Data are from one experiment. Cell counts are indicated in the plots (n=3 wells per antibody combination). Cells were classified into uninfected and infected based on ICP27 expression. Marker intensities were compared using unpaired two-sided Mann-Whitney U test: * $p < 0.05$, ** $p < 0.01$, *** $p < 0.001$, **** $p < 0.0001$, ns = not significant. Boxplots definitions as in Supplementary Fig. 4a.

d-**f** RNAP II phosphorylation and HSV-1 replication compartments (RCs) in HeLa (**d**), A549 (**e**) and BJ (**f**) cells. Cells were infected with HSV-1 (MOI 0.3) and stained for ICP8 and RNAP II S5P or for ICP8 and RNAP II S2P at 12 hpi. Data are from one experiment. ICP8-expressing cells were classified into RC-negative (no ICP8-positive RCs) and RC-positive (at least one ICP8-positive RC) cells. Marker intensities were compared as in **a**. Boxplots definitions as in Supplementary Fig. 4a.
g, **h** ICP8, RNAP II S5P and RNAP II S2P staining of HSV-1-infected A549 (**g**) and BJ (**h**) cells at 12 hpi. Nucleus outlines are indicated in orange. Scale bar, 10 μm.

SUPPLEMENTARY REFERENCES

1. Pietilä, M. K. smFISH of UL19, UL29, EEA1, ERBB2, RELA and HPRT-1 – single-cell feature values. *Mendeley*. http://dx.doi.org/10.17632/sdt339677d.1 (2023).
2. Pietilä, M. K. smFISH + 4i experiment – single-cell feature values and summaries. *Mendeley*. http://dx.doi.org/10.17632/3mwstbcyzv.1 (2023).
3. Pietilä, M. K. MCU data. *Mendeley*. http://dx.doi.org/10.17632/fxbzsgwpg9.1 (2023).
4. Pietilä, M. K. MEK inhibitor – single-cell feature values. *Mendeley*. http://dx.doi.org/10.17632/fnjtkx4js7.1 (2023).
5. Pietilä, M. K. PI3K inhibitor – Western blot images. *Mendeley*. http://dx.doi.org/10.17632/344b25fk2f.1 (2023).
6. Pietilä, M. K. p-Akt and PI3K inhibitor – singe-cell feature values. *Mendeley*. http://dx.doi.org/10.17632/gxv9t4krxg.1 (2023).
7. Pietilä, M. K. p-ERK and innate immunity – single-cell feature values. *Mendeley*. http://dx.doi.org/10.17632/3p635nrr2v.1 (2023).
8. Pietilä, M. K. P bodies in HeLa, A549 and BJ cells – single-cell feature values. *Mendeley*. http://dx.doi.org/10.17632/mg73d32md4.1 (2023).
9. Pietilä, M. K. PCNA and cell cycle – single-cell features. *Mendeley*. http://dx.doi.org/10.17632/jy6gxf7r5z.1 (2023).
10. Pietilä, M. K. RNA Polymerase II – single-cell feature values. *Mendeley*. http://dx.doi.org/10.17632/jprfpjf6ns.1 (2023).
11. Pietilä, M. K. and Ravantti, J. Code for SPS calculations. *Mendeley*. http://dx.doi.org/10.17632/ytvttnr2nn.1 (2023).
12. Battich, N., Stoeger, T. & Pelkmans, L. Image-based transcriptomics in thousands of single human cells at single-molecule resolution. *Nat. Methods* **10**, 1127–1133 (2013).
13. Schindelin, J. *et al.* Fiji: an open-source platform for biological-image analysis. *Nat. Methods* **9**, 676–682 (2012).
14. Berg, S. *et al.* ilastik: interactive machine learning for (bio)image analysis. *Nat. Methods* **16**, 1226–1232 (2019).
15. Gut, G., Herrmann, M. D. & Pelkmans, L. Multiplexed protein maps link subcellular organization to cellular states. *Science* **361**, eaar7042 (2018).
16. Berry, S., Müller, M., Rai, A. & Pelkmans, L. Feedback from nuclear RNA on transcription promotes robust RNA concentration homeostasis in human cells. *Cell Syst.* **13**, 454-470.e15 (2022).
17. Snijder, B. *et al.* Single-cell analysis of population context advances RNAi screening at multiple levels. *Mol. Syst. Biol.* **8**, 579 (2012).
