# Supplementary material for: Cellular state landscape and herpes simplex virus type 1 infection progression are connected
Source: Nat Commun. 2023 Jul 27;14:4515. doi: 10.1038/s41467-023-40148-6 (PMC10374626; doi:10.1038/s41467-023-40148-6)
Supplement: Supplementary file 3 — Description of Additional Supplementary Files [file 41467_2023_40148_MOESM3_ESM.pdf]

Description of Additional Supplementary Files

File Name: Supplementary Data 1
Description: **Related to Methods.** Materials.

File Name: Supplementary Data 2
Description: **Related to Methods.** Statistical information.

File Name: Supplementary Data 3
Description: **Related to Supplementary Fig. 6 and Methods.** Cellular features with Pearson correlation coefficient (r) >=0.3 or <=-0.3.

File Name: Supplementary Movie 1
Description: **Related to Fig. 3a.** UMAP of 242,362 mock- and HSV-1-infected HeLa cells generated from the high-dimensional cellular state space, colored by cell subpopulation displayed at different time points from 1.5 to 12 hpi (mock cells, uninfected cells without infected neighbors, uninfected cells with infected neighbors, and infected cells).

File Name: Supplementary Movie 2
Description: **Related to Fig. 3b, c.** UMAPs of 47,185 uninfected HeLa cells without infected neighbors (left) and 73,871 uninfected cells with infected neighbors (right) generated from the high-dimensional cellular state space, colored by the time point.

File Name: Supplementary Movie 3
Description: **Related to Fig. 3d, e.** UMAPs of 37,730 infected HeLa cells, colored by the time point. Left, projection using cellular features. Right, projection using viral features.
