# Supplementary material for: Cellular state landscape and herpes simplex virus type 1 infection progression are connected
Source: Nat Commun. 2023 Jul 27;14:4515. doi: 10.1038/s41467-023-40148-6 (PMC10374626; doi:10.1038/s41467-023-40148-6)
Supplement: Supplementary file 10 — Reporting Summary [file 41467_2023_40148_MOESM10_ESM.pdf]

nature portfolio

Corresponding author(s): Maija K. Pietilä, Cornel Fraefel

Last updated by author(s): Jun 17, 2023

Reporting Summary

Nature Portfolio wishes to improve the reproducibility of the work that we publish. This form provides structure for consistency and transparency in reporting. For further information on Nature Portfolio policies, see our Editorial Policies and the Editorial Policy Checklist.

Statistics

For all statistical analyses, confirm that the following items are present in the figure legend, table legend, main text, or Methods section.

n/a	Confirmed	
☐	☒	The exact sample size (*n*) for each experimental group/condition, given as a discrete number and unit of measurement
☐	☒	A statement on whether measurements were taken from distinct samples or whether the same sample was measured repeatedly
☐	☒	The statistical test(s) used AND whether they are one- or two-sided<br>*Only common tests should be described solely by name; describe more complex techniques in the Methods section.*
☐	☒	A description of all covariates tested
☐	☒	A description of any assumptions or corrections, such as tests of normality and adjustment for multiple comparisons
☐	☒	A full description of the statistical parameters including central tendency (e.g. means) or other basic estimates (e.g. regression coefficient) AND variation (e.g. standard deviation) or associated estimates of uncertainty (e.g. confidence intervals)
☐	☒	For null hypothesis testing, the test statistic (e.g. *F*, *t*, *r*) with confidence intervals, effect sizes, degrees of freedom and *P* value noted<br>*Give P values as exact values whenever suitable.*
☒	☐	For Bayesian analysis, information on the choice of priors and Markov chain Monte Carlo settings
☐	☒	For hierarchical and complex designs, identification of the appropriate level for tests and full reporting of outcomes
☐	☒	Estimates of effect sizes (e.g. Cohen's *d*, Pearson's *r*), indicating how they were calculated

Our web collection on statistics for biologists contains articles on many of the points above.

Software and code

Policy information about availability of computer code

Data collection	- Olympus cellSens v3.2<br>- TissueMaps v0.6.0: https://github.com/pelkmanslab/TissueMAPS
Data analysis	- Fiji v2.0.0-rc-69/1.52p<br>- Ilastik v1.3<br>- TissueMaps v0.6.0: https://github.com/pelkmanslab/TissueMAPS<br>- Python v2.7.12, v3.9.7, v3.10<br>- Visual Studio Code v1.77.3<br>- Rstudio v1.2<br>- R v3.6.3<br>- napari v0.4.15: https://doi.org/10.5281/zenodo.7276432<br>- popcon v0.1.0 (code for population-context features): https://github.com/scottberry/popcon<br>- Code for MCU calculations: https://github.com/scottberry/mcu<br>- Code for SPS calculations: http://dx.doi.org/10.17632/ytvttnr2nn.1

For manuscripts utilizing custom algorithms or software that are central to the research but not yet described in published literature, software must be made available to editors and reviewers. We strongly encourage code deposition in a community repository (e.g. GitHub). See the Nature Portfolio guidelines for submitting code & software for further information.

Data

Policy information about availability of data

All manuscripts must include a data availability statement. This statement should provide the following information, where applicable:

- Accession codes, unique identifiers, or web links for publicly available datasets
- A description of any restrictions on data availability
- For clinical datasets or third party data, please ensure that the statement adheres to our policy

Source data are provided with this paper (see Supplementary Table 1). Western blot, single-cell, and single-pixel datasets generated during the current study have been deposited at Mendeley, and the DOIs are listed in Supplementary Table 1 and below. Raw microscopy image datasets reported in this paper will be shared by the corresponding author Cornel Fraefel upon reasonable request. Any additional information required to reanalyze the data reported in this paper is available upon request.

Western blot, single-cell and single-pixel datasets are publicly available at Mendeley:
http://dx.doi.org/10.17632/gxv9t4krxg.1
http://dx.doi.org/10.17632/344b25fk2f.1
http://dx.doi.org/10.17632/jprfpjf6ns.1
http://dx.doi.org/10.17632/mg73d32md4.1
http://dx.doi.org/10.17632/fxbzsgwpg9.1
http://dx.doi.org/10.17632/3mwstbcyzv.1
http://dx.doi.org/10.17632/sdt339677d.1
http://dx.doi.org/10.17632/fnjtkx4js7.1
http://dx.doi.org/10.17632/3p635nrr2v.1
http://dx.doi.org/10.17632/jy6gxf7r5z.1

Human research participants

Policy information about studies involving human research participants and Sex and Gender in Research.

Reporting on sex and gender	N/A
Population characteristics	N/A
Recruitment	N/A
Ethics oversight	N/A

Note that full information on the approval of the study protocol must also be provided in the manuscript.

Field-specific reporting

Please select the one below that is the best fit for your research. If you are not sure, read the appropriate sections before making your selection.

☒ Life sciences ☐ Behavioural & social sciences ☐ Ecological, evolutionary & environmental sciences

For a reference copy of the document with all sections, see nature.com/documents/nr-reporting-summary-flat.pdf

Life sciences study design

All studies must disclose on these points even when the disclosure is negative.

Sample size	smFISH experiment of HSV-1 and cellular transcripts was performed using one biological replicate with five technical replicates, and smFISH + 4i experiment was performed using one biological replicate with two (mock infection) or four (HSV-1 infection) technical replicates. Immunofluorescence experiments were performed using 1-2 biological replicates with 1-3 technical replicates. Western blot experiments were performed using 3 biological replicates. No statistical method was used to predetermine sample size, but the number of replicates was chosen based on previous experiments carried out in our laboratories and based our on previous publications: Gut, G., Herrmann, M. D. & Pelkmans, L. Multiplexed protein maps link subcellular organization to cellular states. Science 361, eaar7042 (2018); Kramer, B. A., Sarabia Del Castillo, J. & Pelkmans, L. Multimodal perception links cellular state to decision-making in single cells. Science 377, 642-648 (2022); and Berry, S., Müller, M., Rai, A. & Pelkmans, L. Feedback from nuclear RNA on transcription promotes robust RNA concentration homeostasis in human cells. Cell Syst. 13, 454-470.e15 (2022). The rationale in imaging-based experiments was to collect data from thousands of single cells and in Western blot experiments to have 3 independent experiments.
Data exclusions	In image-based experiments, cells at image borders, missegmented cells and mitotic cells were removed from datasets. In addition, cells with extremely high intensity values were removed by excluding cells that had a mean intensity higher than the 99.995th percentile.
Replication	Imaging-based experiments: Due to the nature of the technique, 4i experiment was performed only once, but with 2 or 4 technical replicates.

Replication	In addition, we compared our 4i data with the literature of known markers in HSV-1 infection to validate findings. All main findings that did not require multiplexing of all cellular markers were also replicated at least 2 times in HeLa cells and successfully reproduced the experimental findings. Additionally, these findings were also successfully reproduced in two other cell lines, A549 and BJ.<br>Western blot experiments: These findings were successfully reproduced 3 times in HeLa cells.
Randomization	No experimental groups were used in this study.
Blinding	Blinding was not relevant in this study because no experimental groups were used.

Reporting for specific materials, systems and methods

We require information from authors about some types of materials, experimental systems and methods used in many studies. Here, indicate whether each material, system or method listed is relevant to your study. If you are not sure if a list item applies to your research, read the appropriate section before selecting a response.

Materials & experimental systems

n/a	Involved in the study	
☐	☒	Antibodies
☐	☒	Eukaryotic cell lines
☒	☐	Palaeontology and archaeology
☒	☐	Animals and other organisms
☒	☐	Clinical data
☒	☐	Dual use research of concern

Methods

n/a	Involved in the study	
☒	☐	ChIP-seq
☒	☐	Flow cytometry
☒	☐	MRI-based neuroimaging

Antibodies

Antibodies used

51 antibodies were used in this study and their information is provided in Supplementary Table 1.

Validation

All antibodies used in this study are commercially available.

Rabbit polyclonal anti-C23, chicken polyclonal anti-H2B, rabbit polyclonal anti-H3K4me3, rat monoclonal anti-RNA polymerase II subunit B1 (phospho CTD Ser-2), and goat polyclonal anti-chicken IgY (H&L) Alexa Fluor 405 are cited in reference Berry, S., Müller, M., Rai, A. & Pelkmans, L. Feedback from nuclear RNA on transcription promotes robust RNA concentration homeostasis in human cells. Cell Syst. 13, 454-470.e15 (2022).

Mouse monoclonal anti-EEA1, mouse monoclonal anti-alpha-tubulin, rabbit polyclonal anti-calreticulin, mouse monoclonal anti-GM130, mouse monoclonal anti-Hsp60, rabbit polyclonal anti-DDX6, rabbit monoclonal anti-phospho-Akt (Ser473), rabbit monoclonal anti-phospho-p44/42 MAPK (ERK) (Thr202/Tyr204), and mouse monoclonal anti-RNA polymerase II CTD repeat YSPTSPS (phospho S5) are cited in reference Kramer, B. A., Sarabia Del Castillo, J. & Pelkmans, L. Multimodal perception links cellular state to decision-making in single cells. Science 377, 642-648 (2022).

Rabbit monoclonal anti-PCNA, mouse monoclonal anti-beta-catenin, mouse monoclonal anti-Nup107, rabbit monoclonal anti-phospho-Akt (Thr308), and rabbit polyclonal anti-TGN46 are cited in reference Gut, G., Herrmann, M. D. & Pelkmans, L. Multiplexed protein maps link subcellular organization to cellular states. Science 361, eaar7042 (2018).

The supplier's homepage provides data (immunofluorescence and Western blot), information concerning species specificity (human, HSV-1) and purity, and/or validation statements, and/or product citations for the following primary antibodies: Rabbit monoclonal anti-4E-BP1, rabbit polyclonal anti-beta-actin (ab8227), mouse monoclonal anti-beta-actin, rabbit monoclonal anti-cleaved caspase-3 (Asp175), goat polyclonal anti-EGFR, rabbit polyclonal anti-H3, goat polyclonal anti-HCFC1, mouse monoclonal anti-ICP0, mouse monoclonal anti-ICP4, rabbit monoclonal anti-IRF3, rabbit monoclonal anti-IRF7, rabbit monoclonal anti-NF-κB p65, rabbit polyclonal anti-NRF2, rabbit monoclonal anti-phospho-EGF receptor (Tyr1068), rabbit polyclonal anti-RNA polymerase II CTD repeat YSPTSPS, mouse monoclonal anti-SRSF2, rabbit polyclonal anti-phospho-STAT1 (Ser727), rabbit monoclonal anti-phospho-STAT1 (Tyr701), and mouse monoclonal anti-VP16.

The supplier's homepage provides data (immunofluorescence), information concerning species specificity (human, HSV-1) and purity, and/or validation statements, and/or product citations for the following primary antibodies: mouse monoclonal anti-ICP5, mouse monoclonal anti-ICP8, mouse monoclonal anti-ICP27, rabbit recombinant Alexa Fluor® 488 Anti-RNA polymerase II CTD repeat YSPTSPS (phospho S5), and rabbit polyclonal anti-SP100.

The supplier's homepage provides data (Western blot), information concerning species specificity (human) and purity, and/or validation statements, and/or product citations for the following primary antibodies: rabbit polyclonal anti-phospho-CDK9 (Thr186).

The supplier's homepage provides data (immunofluorescence), information concerning species specificity and purity, and/or validation statements, and/or product citations for the following secondary antibodies: donkey polyclonal anti-goat IgG (H+L) Alexa Fluor 568, donkey polyclonal anti-mouse IgG (H+L) Alexa Fluor 488, donkey polyclonal anti-mouse IgG (H+L) Alexa Fluor 568, donkey polyclonal anti-rabbit IgG (H+L) Alexa Fluor 488, and donkey polyclonal anti-rabbit IgG (H+L) Alexa Fluor 647.

The supplier's homepage provides data (Western blot), information concerning species specificity and purity, and/or validation

statements, and/or product citations for the following secondary antibodies: Goat polyclonal anti-Mouse IgG (H+L) IRDye 680RD and goat polyclonal anti-Rabbit IgG (H+L) IRDye 800CW.

Eukaryotic cell lines

Policy information about cell lines and Sex and Gender in Research

Cell line source(s)	- Human: lung carcinoma epithelial cell line A549, ATCC CCL-185 (male)<br>- Human: cervical cancer epithelial cell line HeLa Kyoto (female); HeLa Kyoto cells (S. Narumiya, University of Kyoto, Japan; RRID:CVCL_1922) were obtained from J. Ellenberg (EMBL Heidelberg); Battich, N., Stoeger, T. & Pelkmans, L. Image-based transcriptomics in thousands of single human cells at single-molecule resolution. Nat. Methods 10, 1127–1133 (2013)<br>- Human: foreskin fibroblast cell line BJ (male), ATCC CRL-2522<br>- Cercopithecus aethiops: Vero, ECACC 84113001
Authentication	HeLa Kyoto cells have been authenticated by karyotyping (Battich, N., Stoeger, T. & Pelkmans, L. Image-based transcriptomics in thousands of single human cells at single-molecule resolution. Nat. Methods 10, 1127–1133 (2013)).<br>Other cell lines used were not authenticated.
Mycoplasma contamination	A549 cells, HeLa Kyoto cells, BJ cells, and Vero cells were tested negative for mycoplasma.
Commonly misidentified lines<br>(See ICLAC register)	No commonly misidentified cell lines were used in this study.
